# Pre- and postoperative left atrial and ventricular volumetric and deformation analyses in severe aortic regurgitation

**DOI:** 10.1186/s12947-021-00243-4

**Published:** 2021-02-14

**Authors:** Jonas Jenner, Ali Ilami, Johan Petrini, Per Eriksson, Anders Franco-Cereceda, Maria J. Eriksson, Kenneth Caidahl

**Affiliations:** 1 Department of Molecular Medicine and Surgery, Karolinska Institutet, Stockholm, Sweden; 2 Department of Clinical Physiology, Södersjukhuset, Stockholm, Sweden; 3 Department of Clinical Physiology, Karolinska University Hospital, 171 76 Stockholm, Sweden; 4 Division of Cardiovascular Medicine, Department of Medicine Solna, Karolinska University Hospital, Karolinska Institutet, Stockholm, Sweden; 5 Unit of Cardiothoracic Surgery, Karolinska University Hospital, Stockholm, Sweden; 6 Departments of Molecular and Clinical Medicine, Gothenburg University, and Clinical Physiology, Sahlgrenska University Hospital, Gothenburg, Sweden

**Keywords:** Aortic valve replacement, Left atrial function, Transthoracic echocardiography, Left atrial strain

## Abstract

**Background:**

The impact of volume overload due to aortic regurgitation (AR) on systolic and diastolic left ventricular (LV) indices and left atrial remodeling is unclear. We assessed the structural and functional effects of severe AR on LV and left atrium before and after aortic valve replacement.

**Methods:**

Patients with severe AR scheduled for aortic valve replacement (*n* = 65) underwent two- and three-dimensional echocardiography, including left atrial strain imaging, before and 1 year after surgery. A control group was selected, and comprised patients undergoing surgery for thoracic aortic aneurysm without aortic valve replacement (*n* = 20). Logistic regression analysis was used to assess predictors of impaired left ventricular functional and structural recovery, defined as a composite variable of diastolic dysfunction grade ≥ 2, EF < 50%, or left ventricular end-diastolic volume index above the gender-specific normal range.

**Results:**

Diastolic dysfunction was present in 32% of patients with AR at baseline. Diastolic LV function indices and left atrial strain improved, and both left atrial and LV volumes decreased in the AR group following aortic valve replacement. Preoperative left atrial strain during the conduit phase added to left ventricular end-systolic volume index for the prediction of impaired LV functional and structural recovery after aortic valve replacement (model *p* < 0.001, accuracy 70%; addition of left atrial strain during the conduit phase to end-systolic volume index *p* = 0.006).

**Conclusions:**

One-third of patients with severe AR had signs of diastolic dysfunction. Aortic valve surgery reduced LV and left atrial volumes and improved diastolic indices. Left atrial strain during the conduit phase added to the well-established left ventricular end-diastolic dimension for the prediction of impaired left ventricular functional and structural recovery at follow-up. However, long-term follow-up studies with hard endpoints are needed to assess the value of left atrial strain as predictor of myocardial recovery in aortic regurgitation.

## Background

Severe chronic aortic regurgitation (AR) is characterized by volume overload of the left ventricle. This induces structural and functional left ventricular (LV) alterations, which have a negative prognostic impact even in patients who are asymptomatic [1–3].

Despite the emerging potentials of transcatheter valve replacement, the treatment of choice for severe AR is still aortic valve replacement (AVR), with intervention timing based on the presence of symptoms or evidence of increased LV dimensions or systolic dysfunction [4–6]. Recent studies indicate that the cutoff values used in current guidelines might need to be reconsidered [7, 8], and additional measures such as strain imaging for LV have proven useful [9, 10]. Thus, most studies of chronic AR have focused on LV dimensions and systolic function, to intervene while it is still possible to achieve full functional myocardial recovery and optimal life expectancy. However, chronic volume overload in AR also leads to decreased LV relaxation, increased stiffness, and, subsequently increased filling pressures (i.e., diastolic LV dysfunction) [11]. LV diastolic property alterations influence left atrial (LA) size. LA enlargement is an important marker of LV diastolic dysfunction (DD) and is incorporated in echocardiographic DD assessment algorithms [12]. It is still unclear if LA function may be a more sensitive marker of early myocardial impairment and volume overload in chronic severe AR [11].

LA function, in terms of the reservoir, conduit, and contraction phases, can be assessed quantitatively by measuring left atrial strain (LAS) using speckle-tracking echocardiography [13]. LAS has emerged as a useful diagnostic and prognostic parameter in various cardiovascular conditions, including valvular heart disease [14, 15]. However, data on LA phasic function in patients with AR are limited, especially regarding whether this is altered after valve surgery [16]. Furthermore, the value of baseline LA function variables for predicting myocardial recovery after AVR has not been fully elucidated.

We hypothesized that the reduction in LV overload following aortic valve surgery would improve markers of diastolic LV function and LA phasic function. To address this, we performed a prospective, longitudinal study of patients with chronic, severe AR without coronary artery disease using three-dimensional (3D) and two-dimensional (2D) echocardiography, including speckle-tracking echocardiography. We aimed to assess the effects of AR-related LV volume overload on LV and LA structure and function before, and 1 year after, aortic valve surgery. As a secondary aim, we sought to evaluate whether preoperative LA remodeling and functional changes could predict postoperative LV dysfunction.

## Methods

### Study group

The study group was recruited from a prospective, observational study at Karolinska University Hospital in Stockholm, Sweden, conducted from 2007 to 2013. The study included consecutive adult patients free from coronary artery disease (i.e., without symptoms or epicardial stenoses) who were undergoing elective open-heart surgery for aortic valve disease or thoracic aortic aneurysm (TAA) [17]. From this cohort, 101 patients with chronic severe AR and a transvalvular mean pressure gradient < 20 mmHg, with complete 2D and 3D echocardiograms performed pre- and postoperatively, were eligible for inclusion. Exclusion criteria were two or more adjacent LV segments not visualized in either echocardiogram (*n* = 26) or atrial fibrillation (*n* = 5). Five additional cases were excluded for technical reasons (data stored in incompatible format); the final study group consisted of 65 patients.

A control group was included comprising 20 consecutive patients with TAA and no significant aortic valve disease, defined as no or mild AR and transvalvular mean pressure gradient < 20 mmHg, who underwent surgery for TAA during the same period.

### Transthoracic echocardiography

Comprehensive transthoracic 2D and 3D echocardiographic examinations were performed 1 week or less before surgery and at a follow-up visit 1 year after surgery, using commercially available equipment (Philips iE33 or Epic 7; Philips Medical Systems, Bothell, WA, USA). 3D data were acquired over four or seven cardiac cycles, generating full volume datasets. The echocardiograms were performed following current recommendations by two experienced sonographers [18]. Data were stored for offline analysis.

2D echocardiographic data were analyzed using dedicated software (IntelliSpace Cardiovascular 2.3; Philips Medical Systems Nederland B.V., Eindhoven, The Netherlands). LV dimensions and wall thickness were measured in the parasternal long-axis view. LA volume was calculated using the biplane method of disks and indexed to body surface area (BSA). LV stroke work (in gram-meters) was calculated as SW = BP_s_ × SV × 0.014, where BP_s_ is systolic blood pressure and SV is 3D echocardiographic stroke volume [19].

Diastolic function variables were acquired and analyzed according to the current guidelines, and DD was defined by incorporating the ratio between early and late diastolic filling velocities (E/A ratio), the ratio between mitral early filling velocity and average annular tissue velocity (E/e′ ratio), tricuspid regurgitation velocity, and LA volume index (LAVi). Patients were classified into three categories: (i) no or grade 1 DD, (ii) grade 2 DD, and (iii) grade 3 DD [12]. Cases that could not be assigned a DD grade were deemed indeterminate.

LV global longitudinal strain (GLS) was measured using dedicated software (QLab 10.7; Philips Medical Systems Nederland B.V.). The LV wall was traced automatically throughout the cardiac cycle using a speckle-tracking algorithm. The region of interest was manually adjusted if needed. GLS was calculated by averaging the peak systolic longitudinal strain of 17 segments and is expressed as an absolute percentage (|%|).

**Fig. 1:**
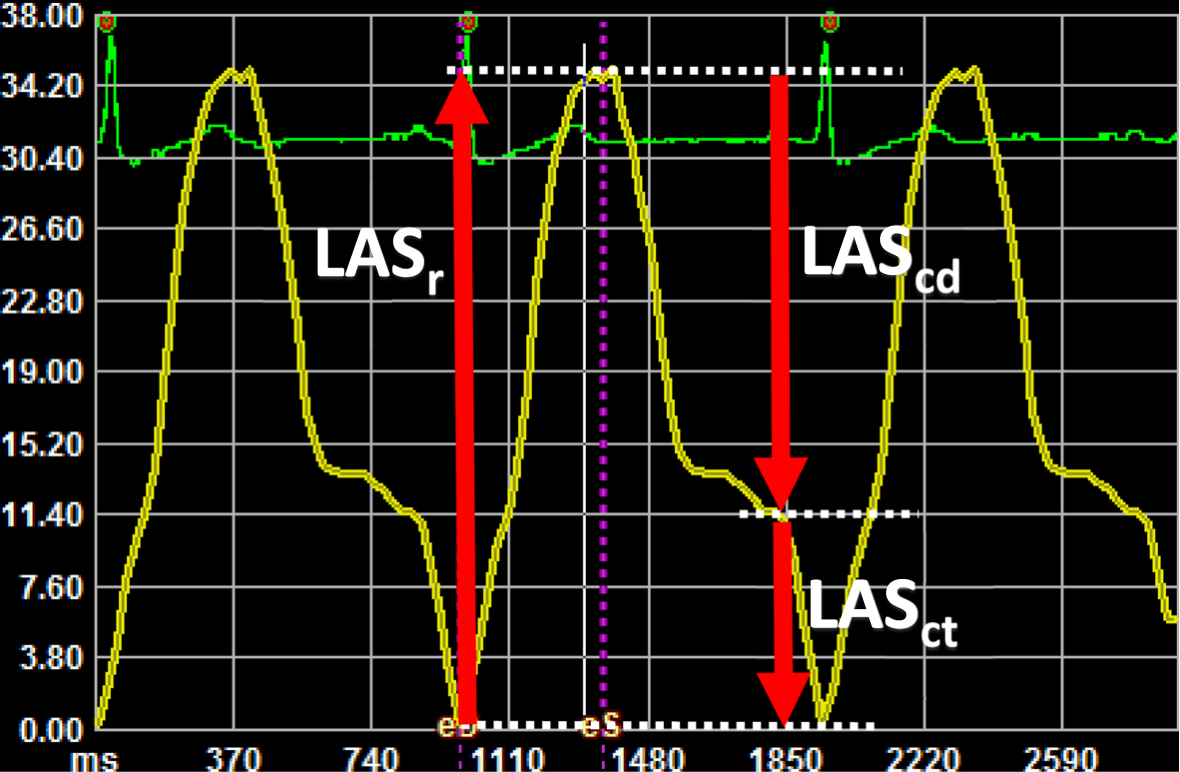
Example of a left atrial strain (LAS) curve (yellow line). The x axis is time, and the y axis represents strain as a percentage. The ECG recording is shown in green for reference. The zero-strain reference is set at end-diastole. Dashed white lines represent strain values at end-diastole (0), end-systole, and the beginning of atrial contraction. Red arrows demonstrate the left atrial strain components. LASr, left atrial reservoir phase; LAScd, left atrial conduit phase; LASct, left atrial contraction phase

LAS was calculated using dedicated software (TomTec-Arena, 2D CPA; TomTec Imaging Systems GmbH, Unterschleißheim, Germany). Two points were placed at the mitral annulus and a third at the LA roof. The LA wall was then automatically tracked through the cardiac cycle, generating an atrial GLS curve. If needed, delineation was corrected manually at end-systole and end-diastole. The zero-strain reference point was set to the end-diastolic frame following mitral valve closure (Fig. 1). The LAS curve was divided into three phases: (i) a reservoir phase (LASr); (ii) a conduit phase (LAScd); and (iii) a contraction phase (LASct) [20]. Results are reported as the means of measurements obtained from four- and two-chamber views.

Intraobserver variabilities in LASr, LASct, and LAScd measurements were assessed by repeated measurements in the same images acquired from 20 randomly selected cases by one blinded observer (A.I.). To determine the interobserver variability, the measurements were repeated in the same images by a second observer (J.J.) who was blinded to the results obtained by the first observer. Agreement was assessed by calculating the intraclass correlation coefficient (*ICC*). *ICC* values for intraobserver agreement of the LASr, LAScd, and LASct estimates were 0.83 (95% CI 0.62–0.93), 0.87 (95% CI 0.69–0.95), and 0.69 (95% C.I. 0.35–0.87), respectively. *ICC* values for interobserver agreement were 0.76 (95% CI 0.43–0.90), 0.85 (95% CI 0.66–0.94), and 0.76 (95% CI 0.19–0.92), respectively.

LV volume and mass measurements in 3D datasets were performed using dedicated software (QLab 10.7, as above). The endocardial surface was outlined using a semi-automated contour detection algorithm, yielding the end-diastolic volume (EDV) and end-systolic volume (ESV). Papillary muscles and trabeculations were included in the cavity. Stroke volume (SV) was calculated from EDV–ESV, and the ejection fraction (EF) was calculated as EDV–ESV/EDV × 100%.

LV mass was calculated using the biplane method of disks in four- and two-chamber views obtained from 3D datasets. The endocardium and epicardium were traced manually in each view, with the papillary muscles included in the LV cavity. The myocardial mass was then calculated by the software. Measurements were made at end-diastole and end-systole, and the LV mass was estimated as the mean of the two measurements and indexed to BSA.

### Surgical procedures

All patients were operated on via a midline sternotomy using cardiopulmonary bypass. In the AR group, AVR was performed using biological valve prostheses (*n* = 16), mechanical valve prostheses (*n* = 15), aortic root bioprostheses (*n* = 5), or aortic root replacement with a mechanical composite graft (*n* = 5). Isolated aortic valve repair was performed in 21 patients, while 3 underwent aortic root replacement with reimplantation of the native valve. The group with isolated TAA received synthetic tubular aortic grafts but no valve surgery.

### N-terminal pro-B-type natriuretic peptide (NTproBNP) analysis

Plasma samples were analyzed using a multiplex immunoassay (Olink Cardiovascular III; Olink Proteomics, Uppsala, Sweden). NTproBNP concentration is reported as normalized protein expression (NPX) units. NPX is a log_2_ scale; hence, an increase of 1 NPX unit means a doubling of protein concentration.

### Statistical analysis

Continuous variables are expressed as the mean ± SD or the median and interquartile range (IQR) depending on the data distribution. A *p* value < 0.05 was considered statistically significant. Correlations between continuous variables were assessed by calculating Pearson’s correlation coefficient *r*. Between-groups comparisons of the means of continuous variables were performed using Student’s *t*-test and analysis of variance (ANOVA). Bonferroni correction was used to adjust for multiple comparisons. Differences between unrelated samples of ordinal data were assessed using the Mann–Whitney *U* test. Relationships between categorical variables were assessed using the chi-squared or Fisher’s exact test.

Simple and multiple logistic regression analyses were performed to assess predictors of impaired LV functional and structural recovery, which was defined as a composite variable comprising one or more of the following echocardiographic characteristics at the follow-up examination: EF < 50%, DD grade ≥ 2, and EDV index (EDVi) greater than the gender-specific normal range [18]. Baseline variables with *p* values < 0.10 in simple logistic regressions were assessed for collinearity and entered pairwise in multiple logistic regression models. The reason for restricting the models to two variables at a time was the risk of overfitting. All combinations were tested, and classification tables and Nagelkerke *R*^2^ (R_N_^2^) values were assessed to rank the models with respect to their predictive performances. The likelihood ratio test (LRT) was used to determine whether a variable contributed significantly to the model. Discriminatory ability was assessed by receiver operating characteristic (ROC) analysis and shown as area under the ROC curve (AUC). Statistical analyses were performed using IBM SPSS Statistics (version 26; IBM Corp., Armonk, NY, USA).

## Results

The baseline characteristics of the AR and control groups are listed in Table 1. Patients with AR had higher NTproBNP levels, and 75% had a New York Heart Association (NYHA) class > I, compared with 10% of the controls (*p* < 0.001). At the follow-up examination, no patient undergoing valve replacement, and 3 patients with valve repair, had moderate residual AR; none of which warranted reoperation. Of these 3 patients, one had EF < 50%. There were no cases of atrial fibrillation at the follow-up examinations.

**Table 1:** Preoperative demographic data

	Aortic regurgitation (*n* = 65)	Controls (*n* = 20)	*p* value
Age (years) median (IQR)	54 (46–63)	59 (49–68)	0.086
Male (n)	56 (86%)	11 (55%)	0.009
Body surface area (m^2^)	2.00 ± 0.19	1.96 ± 0.20	0.46
Body mass index (kg/m^2^)	26.1 ± 4.0	26.3 ± 3.6	0.91
Systolic blood pressure (mmHg)	145 ± 16	134 ± 15	0.011
Diastolic blood pressure (mmHg)	70 ± 11	84 ± 9	< 0.001
Diabetes (n)	1 (1.5%)	1 (5%)	0.77
Hypertension (n)	30 (46%)	10 (50%)	0.80
NYHA functional class			< 0.001
I	16 (25%)	18 (90%)	
II	39 (60%)	2 (10%)	
III	10 (15%)	0	
IV	0	0	
NTproBNP (NPX units)	4.4 ± 1.7	3.52 ± 1.3	0.043
Bicuspid aortic valve (n)	38 (58%)	9 (45%)	0.29

IQR, Interquartile range, NPX units, normalized protein expression units (log_2_ scale), NTproBNP, N-terminal pro-B-type natriuretic peptide; NYHA*,* New York Heart Association

### LV size and function before and after surgery

At baseline, the patients with AR had increased LV dimensions and volumes, larger SV, increased LV mass index (LVMi), and lower EF compared with the controls, whereas no difference in GLS was found (Table 2). In patients with AR, the NTproBNP measure was correlated with GLS (*r =* − 0.43, *p* = 0.001), but not with EF (*p* = 0.076).

**Table 2:** Preoperative and follow-up values for left ventricular and left atrial dimensions, volumes, and function

	Aortic regurgitation (*n* = 65)			Controls (*n* = 20)			Baseline comparison
	Baseline	Follow-up	***p*** value	Baseline	Follow-up	***p*** value	***p*** value
Left ventricular indices							
End-diastolic diameter (mm)	62.6 ± 6	49.3 ± 6	< 0.001	48.5 ± 3	46.2 ± 4	0.005	< 0.001
End-systolic diameter (mm)	44.0 ± 7	34.4 ± 5	< 0.001	33.1 ± 4	32.5 ± 4	0.80	< 0.001
Interventricular septum (mm)	11.7 ± 2.0	12.2 ± 1.7	0.004	11.3 ± 2.0	11.4 ± 1.7	0.71	0.48
Posterior wall thickness (mm)	10.3 ± 1.4	9.5 ± 1.8	0.003	8.9 ± 1.7	9.3 ± 1.6	0.29	< 0.001
End-diastolic volume (mL)	224 ± 62	132.4 ± 36	< 0.001	109 ± 20	111 ± 23	0.25	< 0.001
End-diastolic volume index (mL/m^2^)	113 ± 30	66 ± 17	< 0.001	56 ± 9	57 ± 10	0.40	< 0.001
End-systolic volume (mL)	103 ± 41	57 ± 18	< 0.001	43 ± 8	45 ± 11	0.25	< 0.001
End-systolic volume index (mL/m^2^)	52 ± 20	28 ± 8	< 0.001	22 ± 4	23 ± 5	0.41	< 0.001
Stroke volume (mL)	121 ± 31	76 ± 22	< 0.001	65 ± 14	66 ± 15	0.41	< 0.001
Ejection fraction (%)	55 ± 7.3	57 ± 7.1	0.027^a^	60 ± 4	60 ± 7	0.79	< 0.001
Global longitudinal strain (|%|)	19.0 ± 3.0	19.4 ± 2.5	0.34	19.9 ± 2	19.6 ± 2	0.54	0.24
Left ventricular mass index (g/m^2^)	81 ± 19	62 ± 16	< 0.001	48 ± 11	48 ± 11	0.87	< 0.001
Diastolic function indices							
Mitral E velocity (m/s)	0.76 ± 0.2	0.73 ± 0.2	0.17	0.70 ± 0.2	0.76 ± 0.2	0.14	0.23
Mitral A velocity (m/s)	0.58 ± 0.2	0.59 ± 0.2	0.84	0.63 ± 0.2	0.66 ± 0.2	0.38	0.30
Mitral E/A ratio	1.5 ± 0.7	1.3 ± 0.5	0.063	1.3 ± 0.8	1.2 ± 0.6	0.93	0.17
Mitral E-wave Deceleration time (ms)	205 ± 57	238 ± 52	0.001	212 ± 44	232 ± 52	0.11	0.66
Septal e′ (m/s)	0.074 ± 0.02	0.075 ± 0.02	0.85	0.066 ± 0.02	0.070 ± 0.02	0.46	0.11
Lateral e′ (m/s)	0.097 ± 0.03	0.12 ± 0.03	0.005	0.087 ± 0.02	0.10 ± 0.01	0.071	0.13
Mitral average E/e′ ratio	9.3 ± 3	8.0 ± 3	0.012	10.2 ± 3	9.1 ± 2	0.31	0.30
Tricuspid regurgitation velocity (m/s)	2.5 ± 0.3	2.3 ± 0.4	0.080	2.3 ± 0.2	2.3 ± 0.2	0.33	0.047^a^
Diastolic dysfunction grade							
0 or 1	39 (60%)	55 (85%)	0.003	20 (100%)	18 (90%)	0.30	0.002
2	6 (9%)	2 (3%)		0	1 (5%)		
3	15 (23%)	5 (8%)		0	0		
Indeterminate	5 (8%)	3 (4%)		0	1 (5%)		
Left atrial indices							
Left atrial volume index (ml/m^2^)	38.6 ± 12	32.2 ± 11	< 0.001	26.8 ± 8	27.4 ± 8	0.70	< 0.001
LASr (%)	26.3 ± 6.7	29.0 ± 6.2	0.012	25.7 ± 6.0	25.8 ± 4.7	0.92	0.86
LAScd (%)	15.6 ± 6.1	14.0 ± 4.5	0.037^a^	12.1 ± 3.5	11.9 ± 3.7	0.28	0.081
LASct (%)	11.0 ± 4.7	15.3 ± 5.5	< 0.001	12.8 ± 5.6	13.8 ± 4.2	0.43	0.049^a^

^a^Non-significant after Bonferroni correction at α = 0.017; *e′*, tissue doppler-derived mitral annular early diastolic velocity, *LAScd* Left atrial strain conduit phase, *LASct* Left atrial strain contraction phase, *LASr* Left atrial strain reservoir phase

**Fig. 2:**
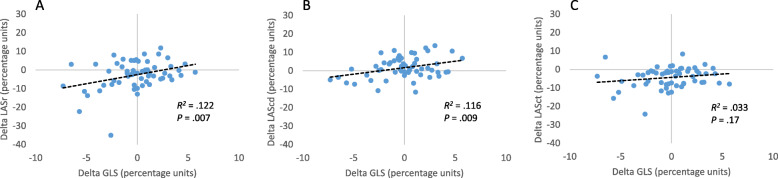
Changes between baseline and follow-up in left ventricular and left atrial strain indices in patients with aortic regurgitation. Left ventricular global longitudinal strain (GLS) vs. left atrial strain (LAS) reservoir phase (**a**), conduit phase (**b**), and contraction phase (**c**). GLS, Global longitudinal strain; LASr, left atrial reservoir phase; LAScd, left atrial conduit phase; LASct, left atrial contraction phase

At follow-up, decreases were observed in LV dimensions and volumes, SV, wall thickness, and LVMi, while the EF had increased in patients with AR; these variables were unchanged in the control group. The change in GLS from baseline to follow-up in patients with AR was correlated with the changes in LASr (*r =* 0.35, *p* = 0.007) and LAScd (*r =* 0.34, *p* = 0.009; Fig. 2).

### LV diastolic function and LA function before and after surgery

At baseline, patients with AR had larger LAVi, a higher prevalence of DD grade 2 or 3, and lower LASct measures compared with the control group (Table 2). NTproBNP correlated with LASr (*r =* − 0.43, *p* = 0.001), LAScd (*r =* − 0.39, *p* = 0.002), and LAVi (*r =* 0.47, *p* < 0.001).

**Fig. 3:**
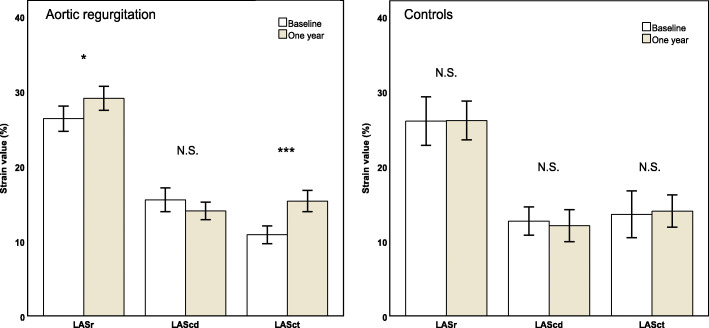
Left atrial strain (LAS) components at baseline and 1 year after surgery. Error bars represent 95% confidence intervals. LASr, left atrial reservoir phase; LAScd, left atrial conduit phase; LASct, left atrial contraction phase. ^*^p ≤ 0.05, ^***^p ≤ 0.001, N.S. *p* > 0.05

At follow-up in the AR group, indices of LV diastolic function improved, LAVi decreased, and increases were seen in the LASr and LASct estimates. No changes were observed in the control group in diastolic function, LAVi, or LAS variables (Table 2; Fig. 3).

Patients with AR classified as DD grades 2 or 3 had lower LASr, LASct, and LASct/LASr ratios, and higher NTproBNP levels compared with patients with AR in DD grades 0 or 1 and with controls (Table 3).

**Table 3:** Left atrial strain and NTproBNP levels in patients with aortic regurgitation (based on diastolic dysfunction grade) and controls

	Aortic regurgitation (*n* = 60)		Controls (*n* = 20)	Overall *p* value
	DD grades 0 or 1 (***n*** = 39)	DD grades 2 or 3 (***n*** = 21)		
LASr (%)	27.9 ± 5.1^*^	23.8 ± 7.8	25.7 ± 6.0	0.030
LAScd (%)	15.3 ± 6.8	15.8 ± 6.8	12.1 ± 3.5	0.26
LASct (%)	12.6 ± 4.2^*^	8.0 ± 3.7^†^	12.8 ± 5.6	< 0.001
LASct/LASr ratio	0.47 ± 0.15^*^	0.34 ± 0.13^†^	0.50 ± 0.14	0.001
NTproBNP (NPX units)	3.79 ± 1.61^*^	5.03 ± 1.54^†^	3.53 ± 1.31	0.002

*DD* Diastolic dysfunction, *LAScd* Left atrial conduit phase, *LASct* Left atrial contraction phase, *LASr* Left atrial reservoir phase, *NPX units* Normalized protein expression units (log_2_ scale), *NTproBNP* N-terminal pro-B-type natriuretic peptide. ^*^p < 0.05 for the comparison between DD grades 0 or 1 and DD grades 2 or 3, ^†^*p* < 0.05 for the comparison between DD grades 2 or 3 and the control group

### Determinants of impaired LV functional and structural recovery

At follow-up, 27 patients fulfilled one or more of the composite outcome criteria: EF < 50%, DD grade ≥ 2, EDVi above the normal range according to current guidelines (> 79 mL/m^2^ for men and < 71 mL/m^2^ for women) [18]. Baseline variables were analyzed using simple logistic regression (Table 4). The variable that best predicted impaired LV functional and structural recovery in the unadjusted analysis was end-systolic volume index (ESVi) (accuracy 69%; OR 1.07, *p* = 0.001). Variables with *p* values < 0.10 in simple regressions were tested for collinearity and subsequently added to ESVi in multiple regression analyses. The model that best predicted impaired LV functional and structural recovery was LAScd combined with ESVi (accuracy 70%, Table 5). LAScd was the only variable that added significantly to ESVi to predict the outcome variable (LRT *p* = 0.006); the model with LAScd and ESVi had greater discriminatory ability compared with ESVi alone (AUC 0.83 vs. 0.78, *p* = 0.046).

**Table 4:** Simple logistic regression analyses of predictors of impaired LV functional and structural recovery in aortic regurgitation patients undergoing aortic valve surgery

	Odds ratio (95% CI)	*p* value	*R*_N_^2^
Age	0.96 (0.92–1.00)	0.045	0.09
Gender	0.6 (0.14–2.67)	0.51	0.10
EDVi	1.04 (1.02–1.07)	0.001	0.29
ESVi	1.07 (1.03–1.12)	0.001	0.28
EF	0.93 (0.86–1.01)	0.091	0.07
GLS	0.9 (0.75–1.08)	0.26	0.03
Stroke work	1.01 (1–1.02)	0.040	0.10
DD grade ≤ 1 vs. ≥ 2	0.62 (0.2–1.89)	0.40	0.02
E/e′	0.94 (0.82–1.08)	0.40	0.02
LAVi	0.99 (0.94–1.03)	0.53	0.01
LASr	1.1 (1–1.2)	0.059	0.05
LAScd	1.15 (1.03–1.28)	0.010	0.17
LASct	0,95 (0,84 – 1,07)	0.36	0.02
NYHA I vs. II–III	1.68 (0.52–5.43)	0.38	0.02
NTproBNP	0.98 (0.72–1.33)	0.91	0.0

*CI* Confidence interval, *DD* Diastolic dysfunction, *E/e′* ratio between mitral early filling velocity and annular tissue velocity, *EF* Ejection fraction, *EDVi* End-diastolic volume index, *ESVi* End-systolic volume index, *GLS* Left ventricular global longitudinal strain, *LAScd* Left atrial strain conduit phase, *LASct* Left atrial strain contraction phase, *LASr* Left atrial strain reservoir phase, *LAVi* Left atrial volume index, *NTproBNP* N-terminal pro-B-type natriuretic peptide, *NYHA* New York Heart Association functional class, *R*_*N*_^*2*^ Nagelkerke *R*^2^

**Table 5:** Multiple logistic regression analysis and LRT showing models that best predicted impaired LV functional and structural recovery in patients undergoing surgery for aortic regurgitation

	Model *p*	Accuracy (%)	Sensitivity (%)	Specificity (%)	*R*_N_^2^	AUC (95% CI)	LRT *p*
ESVi	< 0.001	69	59	77	0.28	0.78 (0.66–0.88)	
*Variable added to ESVi*							
**Age**	< 0.001	69	59	77	0.33	0.78 (0.66–0.90)	0.11
**LAScd**	< 0.001	70	65	74	0.42	0.83 (0.72–0.93)	0.006
**LASr**	< 0.001	68	61	74	0.33	0.80 (0.69–0.91)	0.10
**Stroke work**	0.001	68	56	77	0.29	0.77 (0.65–0.89)	0.70

*AUC* Area under the ROC curve, *ESVi* End-systolic volume index, *LAScd* Left atrial strain conduit phase, *LASr* Left atrial strain reservoir phase, *LRT* likelihood ratio test, *R*_*N*_^*2*^, Nagelkerke *R*^2^, *Zva* Valvulo-arterial impedance

## Discussion

To the best of our knowledge, this is the first follow-up study to assess changes in LA phasic function following AVR in patients with severe AR. Our results demonstrate that patients with AR have larger LAVi and reduced LASct compared with controls. One year after surgery, LAVi and LAScd had decreased, LA strain during LASr and LASct had increased, and an improvement was seen in diastolic function indices and consequently DD grade. These findings suggest that the volume overload imposed by AR affects not only LV volume and mass and systolic function but also LV diastolic properties and LA structure and function. We also found that preoperative LA function, in terms of LAScd, added to the established measure of end-systolic LV dimension for the prediction of impaired LV functional and structural recovery following AVR for severe AR.

### Systolic LV function

Severe AR leads to increased preload of the LV, inducing progressive dilation, which in turn increases wall stress and, over time, can cause structural myocardial changes and depressed ventricular systolic function [21]. Thus, LV size and EF are well-established predictors of disease progression and outcome in patients with AR [1, 4, 5, 22, 23]. The EF of the AR group in our study increased from baseline to follow-up. This EF increase was a function of reductions in EDV, ESV, and SV, and might primarily be a marker of reversed LV remodeling in these patients. We also measured GLS, which has previously been shown predictive of mortality, the need for surgery, and LV systolic dysfunction in patients with AR [9, 10, 24, 25]. We found baseline average GLS values in the same range (i.e., around − 19%) as those reported in a recent study of patients with AR, where lower absolute values were found to be associated with increased mortality [9]. However, we did not find an association between GLS and the composite outcome of impaired LV functional and structural recovery following surgery. Furthermore, GLS did not change significantly from baseline to follow-up. This may be partly explained by the load dependency of GLS, where an increase in SV and concomitant increase in EDV, as seen in AR, will have opposing effects on GLS [26, 27]. The same observation was made by Vollema et al., who suggested that the finding was related to the Frank–Starling mechanism; hence, a reduction in preload will lead to a decrease in myocardial contraction force and consequently GLS [28]. The interaction between GLS and loading conditions was also evident in a study of 47 patients with AR undergoing valve surgery, in whom the absolute GLS decreased after surgery, whereas the normalized GLS calculated as the GLS/EDV ratio increased, primarily driven by a reduction in EDV after surgery [29].

We further analyzed the relationship between changes in LAS and GLS, finding that the latter had a moderate linear correlation with changes in LASr and LAScd. This finding confirms that GLS and LAS partly interact during systole, probably because systolic atrioventricular plane movement affects LA strain in segments close to the mitral annulus.

### Diastolic LV function and LA function

LV–LA interaction is complex in patients with AR. If the LV is compliant, increased preload results in increased EDV without any increase in diastolic LV filling pressure. In the latter disease stages, there is an increase in the fibrous content of the LV myocardium, leading to increased stiffness, which contributes to increased LV filling pressure and a subsequent increase in mean LA pressure and LA volume; at some point, this will become associated with symptom development [30, 31]. Previous studies have revealed a significant interaction between LA phasic function and LV diastolic and systolic function in patients with aortic stenosis, coronary artery disease, and heart failure, but not in severe AR. In cases of aortic stenosis, the LAScd was correlated with indices of increased LV filling pressure, while LASct was correlated with aortic valve area [32]. In patients with coronary artery disease and heart failure, LASr—and to a lesser extent, LASct—correlate with LV end-diastolic pressure, LA pressure, and NTproBNP levels [32–35]. This is consistent with our findings in patients with AR, where LASr and LAScd correlated with NTproBNP levels.

In a previous study of patients with DD and preserved EF, mild DD was associated with reductions in LAScd and LASr and an increase in LASct, whereas all components of LA phasic function were reduced in patients with higher degrees of DD [36]. We also found an association between LA phasic function and DD in patients with AR, with lower LASr and LASct values in patients with DD grades 2 or 3 compared with those with DD grades 0 or 1. In parallel, the NTproBNP concentration was 2.3 times higher in our patients with DD grades 2 or 3, suggesting that there was, indeed, a difference in LV filling pressure between the groups.

The reduced preoperative LASct in our patients with AR with DD grade >1 indicates a diminished active atrial contraction, consistent with a previous report showing that LA contraction force is lower in patients with AR than in those with aortic stenosis [37]. In a mixed group of patients with AR or aortic stenosis, LASct was the first of the phasic LA function components to be altered in moderate valvular disease, whereas both LASct and LASr were reduced in patients with severe aortic valvular disease with pulmonary hypertension [38]. The reduction in LA contractile function in patients with AR might be explained by an increased afterload imposed on the left atrium caused by a combination of competitive filling of the left ventricle from the aorta and increased stiffness of the LV wall. LA phasic function is also directly influenced by preload, as demonstrated in a study of healthy volunteers, where all LAS components were reduced following an acute preload reduction [39]. The reduction in volume load and LV preload following aortic valve surgery improved LAS components in our patients with AR. The variable that best predicted the composite outcome of impaired LV recovery was the well-established measure of end-systolic LV dimension [4]. However, LAScd added significantly to the prediction, suggesting that LAScd has additive prognostic value for patients with AR. A high prevalence of DD in patients with severe AR, and its adverse impact on postoperative cardiac function, has been reported previously [40]. In our study, moderate or advanced DD was found at baseline in one-third of the patients with AR. Although DD improved postoperatively, DD grades 2 or 3 persisted in some patients. This observation may be related to residual LV fibrosis in these patients. In a previous study, myocardial fibrosis and invasively assessed LV diastolic stiffness were increased in AR patients preoperatively, and remained increased after AVR [41].

### Limitations

The echocardiographic assessment of LV diastolic function and filling pressure in patients with AR remains challenging. There are limited data regarding the accuracy of current criteria for the assessment of increased LV filling pressures in these patients [12]. Nevertheless, in our patients with AR, a significant difference was observed in the NTproBNP concentration in patients with DD grades 2 or 3 compared with patients with DD grades 0 or 1, supporting the validity of the integrative approach to differentiate between normal and increased filling pressures.

Our control group consisted of patients free from significant aortic valve disease, who underwent open thoracic surgery for TAA. Our goal was to assess whether cardiac surgery per se would induce changes in LV function or LAS. Although we cannot exclude small changes in loading conditions due to the surgical correction, we could not demonstrate any significant changes in LV or LA volumes or function in the control group. Therefore, we believe that the volumetric and functional changes observed in our patients with AR were not significantly affected by the trauma of surgery per se*.*

Our relatively short follow-up time precluded use of hard outcomes such as mortality or admissions for recurrent heart failure. Furthermore, we used a composite variable to define impaired LV function and structure, which may limit the generalizability of the results. Thus, our study should be considered a hypothesis-generating one. The prognostic implications of LAS in patients with AR warrants validation with larger cohorts with additional clinical outcome variables.

## Conclusions

One-third of patients with chronic severe AR had signs of impaired LV diastolic function. Aortic valve surgery improved diastolic LV function, decreased LV and LA volumes, and increased LA reservoir and contractile function. We found that LA strain components have an incremental prognostic value to the well-established LV end-systolic dimension for the prediction of impaired LV functional and structural recovery following aortic valve surgery. However, further research in this area with larger, longer-term follow-up studies with hard endpoints will be needed to establish the role of LAS in the preoperative evaluation of patients with AR.

## Data Availability

The data that support the study findings are available from the corresponding author upon reasonable request.

## Abbreviations

ANOVA: Analysis of variance
AR: Aortic regurgitation
AUC: Area under the curve
AVR: Aortic valve replacement
BSA: Body surface area
CI: Confidence interval
DD: Diastolic dysfunction
EDV: End-diastolic volume
ESV: End-systolic volume
EF: Ejection fraction
GLS: Global longitudinal strain
ICC: Intraclass correlation coefficient
LA: Left atrial
LAS: Left atrial strain
LAScd: Left atrial strain conduit phase
LASct: Left atrial strain contraction phase
LASr: Left atrial strain reservoir phase
LAVi: Left atrial volume index
LRT: Likelihood ratio test
LV: Left ventricular
LVMi: Left ventricular mass index
NYHA: New York Heart Association
NPX: Normalized protein expression
NTproBNP: N-terminal pro-B-type natriuretic peptide
ROC: Receiver operating characteristic
R_N_^2^: Nagelkerke *R*^2^
SV: Stroke volume
TAA: Thoracic aortic aneurysm
Zva: Valvulo-arterial impedance
